# Expression of the AcrAB Components of the AcrAB-TolC Multidrug Efflux Pump of *Yersinia enterocolitica* Is Subject to Dual Regulation by OmpR

**DOI:** 10.1371/journal.pone.0124248

**Published:** 2015-04-20

**Authors:** Adrianna Raczkowska, Joanna Trzos, Olga Lewandowska, Marta Nieckarz, Katarzyna Brzostek

**Affiliations:** Department of Applied Microbiology, Institute of Microbiology, Faculty of Biology, University of Warsaw, Warsaw, Poland; Centre National de la Recherche Scientifique, Aix-Marseille Université, FRANCE

## Abstract

OmpR is a transcriptional regulator implicated in the control of various cellular processes and functions in Enterobacteriaceae. This study was undertaken to identify genes comprising the OmpR regulon in the human gastrointestinal pathogen *Yersinia enterocolitica*. Derivatives of an *ompR*-negative strain with random transposon insertions creating transcriptional fusions with the reporter gene *lacZ* were isolated. These were supplied with the wild-type *ompR* allele *in trans* and then screened for OmpR-dependent changes in *β*-galactosidase activity. Using this strategy, five insertions in genes/operons positively regulated by OmpR and two insertions in genes negatively regulated by this protein were identified. Genetic analysis of one of these fusion strains revealed that the gene *acrR*, encoding transcriptional repressor AcrR is negatively regulated by OmpR. Differential analysis of membrane proteins by SDS-PAGE followed by mass spectrometry identified the protein AcrB, a component of the AcrAB-TolC multidrug efflux pump, as being positively regulated by OmpR. Analysis of the activity of the *acrR* and *acrAB* promoters using *gfp* fusions confirmed their OmpR-dependent repression and activation, respectively. The identification of putative OmpR-binding sites and electrophoretic mobility shift assays confirmed that this regulator binds specifically to both promoter regions with different affinity. Examination of the activity of the *acrR* and *acrAB* promoters after the exposure of cells to different chemicals showed that bile salts can act as an OmpR-independent inducer. Taken together, our findings suggest that OmpR positively controls the expression of the AcrAB-TolC efflux pump involved in the adaptive response of *Y*. *enterocolitica* O:9 to different chemical stressors, thus conferring an advantage in particular ecological niches.

## Introduction

Multidrug efflux pumps are major determinants of drug resistance in bacteria. These pumps are classified into five different families according to their structure and function: MFS (major facilitator superfamily), SMR (small multidrug resistance family), MATE (multidrug and toxic compound extrusion family), ABC (ATP binding cassette superfamily), and RND (resistance nodulation cell division family) [1]. The AcrAB-TolC efflux pump belongs to the RND family, members of which are particularly effective in conferring drug resistance in Gram-negative bacteria [2,3]. The AcrAB-TolC efflux pump has a wide substrate spectrum encompassing antibiotics, dyes, detergents, bile salts, toxins and environmental compounds [4,5,6]. AcrAB-TolC is a tripartite system that mediates the expulsion of periplasmic substrates across the outer membrane. AcrB is an inner membrane efflux protein extended into the periplasm, AcrA is a periplasmic adaptor protein and TolC forms a channel in the outer membrane [5,7]. In *Escherichia coli* the expression of the *acrAB* operon is regulated by three activators, MarA, SoxS and Rob, and one repressor, AcrR [8,9,10]. In *Salmonella*, an additional activator, RamA is also involved in the control of *acrAB* transcription [11,12]. Factors that induce the AcrAB-TolC efflux pump in *Salmonella* and *E*. *coli* include indole, bile salts, ethanol, high osmolarity and the stationary phase [8,13]. The AcrAB-TolC efflux pump has yet to be extensively studied in yersiniae species pathogenic to humans. Comparative analysis of clinical strains of *Y*. *enterocolitica* has demonstrated AcrAB and MarA overexpression, which is associated with the fluoroquinolone and multidrug resistance phenotypes [14].

*Yersinia enterocolitica* causes yersiniosis, an infectious disease of the gastrointestinal tract which, after salmonellosis and campylobacteriosis, is the third most common zoonotic bacterial disease in Europe [15]. *Y*. *enterocolitica* is a heterogeneous species that encompasses many bio-serotypes displaying varying degrees of virulence. These bacteria are free-living in the environment or live in association with a mammalian host. Thus, like other enteropathogens, they are exposed to various environmental factors characterizing specific ecological niches [16,17].

Bacterial adaptation to new conditions requires efficient modulation of gene expression. Two-component signal-transduction systems (TCSs) combine signal recognition, signal transduction and gene expression [18]. The prevalence of TCSs is due to their important role in the regulation of bacterial cellular processes including competence, conjugation, sporulation, bioluminescence, antibiotic synthesis, motility, biofilm formation, pathogenesis, regulation of metabolic pathways and transport of nutrients and ions [19]. TCSs enable a rapid response to changes in environmental conditions such as pH, temperature, osmotic pressure, nutrient availability and ion concentrations [18].

The EnvZ/OmpR system of *E*. *coli*, which is responsible for regulating the synthesis of the outer membrane porins OmpF and OmpC, enables cells to survive fluctuations in the osmolarity of the environment [20]. The two components of this system are the transmembrane histidine kinase EnvZ and its cognate response regulator OmpR, a cytoplasmic transcription factor [21]. Further studies have shown that OmpR can act as a global transcriptional regulator involved in controlling the expression of a wide variety of genes in Enterobacteriaceae, including virulence genes of pathogenic strains [22,23,24]. OmpR has been shown to be the most important two-component regulator in the acid response in *Salmonella enterica* sv. Typhimurium, *E*. *coli* and *Y*. *pseudotuberculosis* [25,26,27]. OmpR regulates a type VI secretion system in *Y*. *pseudotuberculosis*, which permits survival in acidic environments by maintaining intracellular pH homeostasis [28].

In *Y*. *enterocolitica*, the EnvZ/OmpR system was first characterized during an analysis of the physiological consequences of the loss of the OmpR protein. An *ompR* mutant (AR4, Δ*ompR*::Km) of *Y*. *enterocolitica* strain Ye9 (bio-serotype 2/O9) subspecies *palearctica*, showed significant sensitivity to osmolarity, oxidative, thermal and acid stress. It was found that OmpR is involved in protecting cells against adverse intracellular conditions experienced following macrophage phagocytosis [29]. OmpR participates in the expression of OmpC, OmpF and OmpX porins, and also important pathogenicity determinants like the Yop proteins, invasin, adhesin Ail and flagella [29,30,31,32,33,34]. Moreover, OmpR appears to modulate the adhesion and invasion abilities of *Y*. *enterocolitica* and promotes biofilm development [35].

In light of the available evidence we assume that OmpR acts as a global transcriptional regulator in *Yersinia* cells. The present study was undertaken to identify genes comprising the OmpR regulon. OmpR-dependent genes were recognized by randomly inserting the *lacZ* reporter gene throughout the genome of an *ompR* mutant of *Y*. *enterocolitica* Ye9, and then comparing β-galactosidase activity in the presence and absence of *ompR* expression. In parallel, proteins from outer membrane protein-enriched fractions, whose synthesis is up- or downregulated by OmpR were also identified. Our results indicate that *acrR* and *acrAB* are members of the OmpR regulon in *Y*. *enterocolitica*. OmpR appears to up-regulate expression of the AcrAB-TolC efflux pump directly by activating *acrAB* transcription and indirectly by inhibiting *acrR* (encoding repressor AcrR) transcription.

## Results

### Identification of OmpR-regulated genes in the genome of *Y*. *enterocolitica*

**Fig 1:**
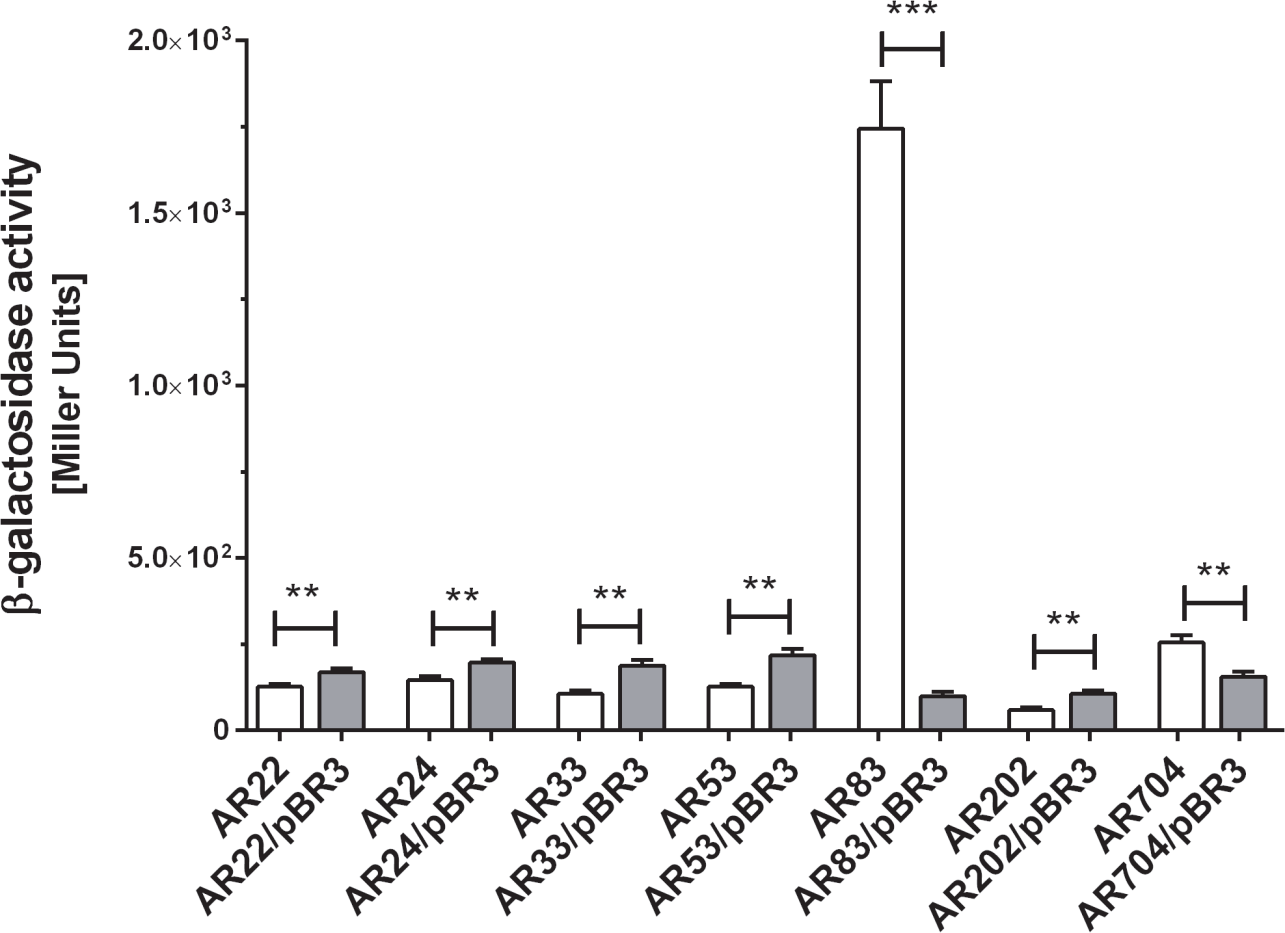
β-galactosidase activity exhibited by different transposon mutants and *trans*-complemented strains. The data represent mean values (± standard deviation) from three independent experiments performed in triplicate. Asterisks indicate statistically significant differences (**—*p*<0.01; ***—*p*<0.001) according to Student’s unpaired *t*-test.

To identify genes whose expression is under the control of the EnvZ/OmpR signaling pathway in *Y*. *enterocolitica* Ye9, we obtained 960 independent chromosomal transcriptional fusions with the *lacZ* reporter gene following transposon mutagenesis of an *ompR* mutant with Tn5-B22. Next, a plasmid expressing *ompR* was introduced into each of these strains using a mass-mating technique. Individual transposon mutants were screened on LB agar containing X-Gal for differences in reporter gene expression with and without *ompR* supplied *in trans*. Of the 960 mutants screened, seven contained insertions in OmpR-regulated genes (Table 1). Five mutants carried insertions in genes/operons positively regulated by OmpR, and two mutants had an insertion in genes negatively regulated by OmpR. Quantitative β-galactosidase assays confirmed the mutant phenotypes and indicated that OmpR increased expression in mutants AR22, AR24, AR33, AR53 and AR202 by between 30 and 80%, while it decreased the expression level in mutant AR704 by almost 40%. In the case of strain AR83, the presence of OmpR *in trans* led to a significant decrease (8-fold) in the expression of the *lacZ* reporter gene (Fig 1).

**Table 1:** Characterization of the sites of Tn5-B22 transposition in *Y*. *enterocolitica* mutants.

Mutant	site of transposon insertion^a^	protein (function)	regulation^d^
AR22	YE105_C2078^b^	TyrR (DNA-binding transcriptional	+
		regulator)	
AR24	YE105_C1595^b^	FimA (pilus assembly protein)	+
AR33	YE105_C0917^b^	general secretion pathway protein J	+
		(type II secretion system)	
AR53	YE105_C3385^c^	glycosyl hydrolase family protein	+
AR83	YE105_C1163^b^	DsrE (oxidative sulfur metabolism)	-
AR202	intergenic region	hypothetical proteins (unknown	+
	(YE105_C2160,	function)	
	YE105_C2160) ^c^		
AR704	YE105_C2117^b^	hypothetical protein (homolog of	-
		tellurite resistance protein TerB)	

^a^ sequence of region flanking transposon insertion comes from the genome of *Y*. *enterocolitica* subsp. *palearctica* 105.5R(r) (NC_015224) deposited in the NCBI database.

^b^ *lacZ* is in the same orientation as the region with similarity to the database entry.

^c^ *lacZ* is in the opposite orientation to the region with similarity to the database entry.

^d^ + or-mean positive or negative OmpR-dependent regulation.

To identify the sites of transposon insertion in the selected mutants, the DNA regions flanking these insertions were amplified by AP-PCR and then sequenced. Analysis of these nucleotide sequences revealed that each mutant was the result of a unique insertion event (Table 2). Five transposon insertions were positioned such that the *lacZ* reporter gene was oriented in the same direction as an open reading frame (ORF) identified by sequence similarity. BLAST searches revealed identity between these ORFs and sequences from *Y*. *enterocolitica* subsp. *palearctica* 105.5R(r). In strain AR22 (positive effect of OmpR) transposition occurred at gene YE105_C2078, encoding DNA-binding transcriptional regulator TyrR. Insertion in strain AR24 (positive effect) was in gene YE105_C1595, which is probably involved in fimbrial synthesis. This gene encodes a homolog of the FimA pilus assembly protein. Strain AR33 (positive effect) had an insertion in gene YE105_C0917, which probably forms an operon with YE105_C0916. These two genes respectively encode general secretion pathway proteins J and I, which are involved in the formation of a type II secretion system. Transposition in strain AR83 (negative effect of OmpR) occurred in gene YE105_C1163, encoding a protein which displays significant similarity to DsrE, an essential factor in oxidative sulfur metabolism. Notably, gene YE105_C1162, encoding transcriptional repressor AcrR was identified immediately upstream of *dsrE*. In strain AR704 (negative effect) the *lacZ* reporter gene was transcribed from the promoter of gene YE105_C2117, encoding a hypothetical protein with homology to the tellurite resistance protein TerB. In the case of strain AR202 (positive effect of OmpR) insertion occurred in the intergenic region between genes YE105_C2160 and YE105_C2161. However, it seems that the reporter gene is transcribed from the promoter of the downstream gene YE105_C2163, encoding hypothetical protein ADZ42659.1 of unknown function. In strain AR53 (positive effect) transposition occurred in gene YE105_C3385, but in the opposite orientation to this gene promoter. Using the BPROM program for the prediction of bacterial promoters (http://linux1.softberry.com/), we identified two hypothetical promoter sequences located between genes YE105_C3382 and YE105_C3383. Thus, the OmpR-dependent regulation in mutant AR53 may be due to transcription from an OmpR-regulated promoter downstream from and oriented convergently to the *lacZ* gene.

**Table 2:** Putative OmpR-regulated proteins of *Y*. *enterocolitica* Ye9 identified by SDS-PAGE and LC-MS/MS^a^.

Putative OmpR	band	protein	gene	theoretical
regulation				molecular
				mass (kDa)^b^
Induction at 27°C	p120	AcrB (inner membrane	YE3101 *Y*. *enterocolitica*	113.36
		protein, component of the	subsp. *enterocolitica* 8081	
		AcrAB-TolC multidrug		
		efflux pump)		
	p110	ferrichrome-iron receptor	Y11_35641	78.57
			*Y*. *enterocolitica* subsp.	
			*palearctica* Y11	
Induction at 37°C	p100	dehydratase (bifunctional	YE105_C0819	93.43
		aconitate hydratase 2/2-	*Y*. *enterocolitica* subsp.	
		methylisocitrate	*palearctica* 105.5R(r)	
		dehydratase)		
	p85	putative ABC transporter ATP-binding	Ye3942 *Y*. *enterocolitica* subsp.	71.91
		protein	*enterocolitica* 8081	
Repression at	p80	60-kDa heat shock protein	YE0354 *Y*. *enterocolitica*	57.67
37°C			subsp.	
			*enterocolitica* 8081	
	p60	elongation factor Tu	YE3927 *Y*. *enterocolitica*	43.22
			subsp.	
			*enterocolitica* 8081	

^a^ Outer membrane protein-enriched fractions from bacteria grown to stationary phase at 27°C and 37°C were analyzed.

^b^ Theoretical molecular masses of *Y*. *enterocolitica* proteins were calculated from their amino acid sequences deposited in the NCBI database using ExPASy proteomics tool Compute pI/Mw (http://www.expasy.org).

### Identification of OmpR-regulated membrane proteins by SDS-PAGE and mass spectrometry analysis

**Fig 2:**
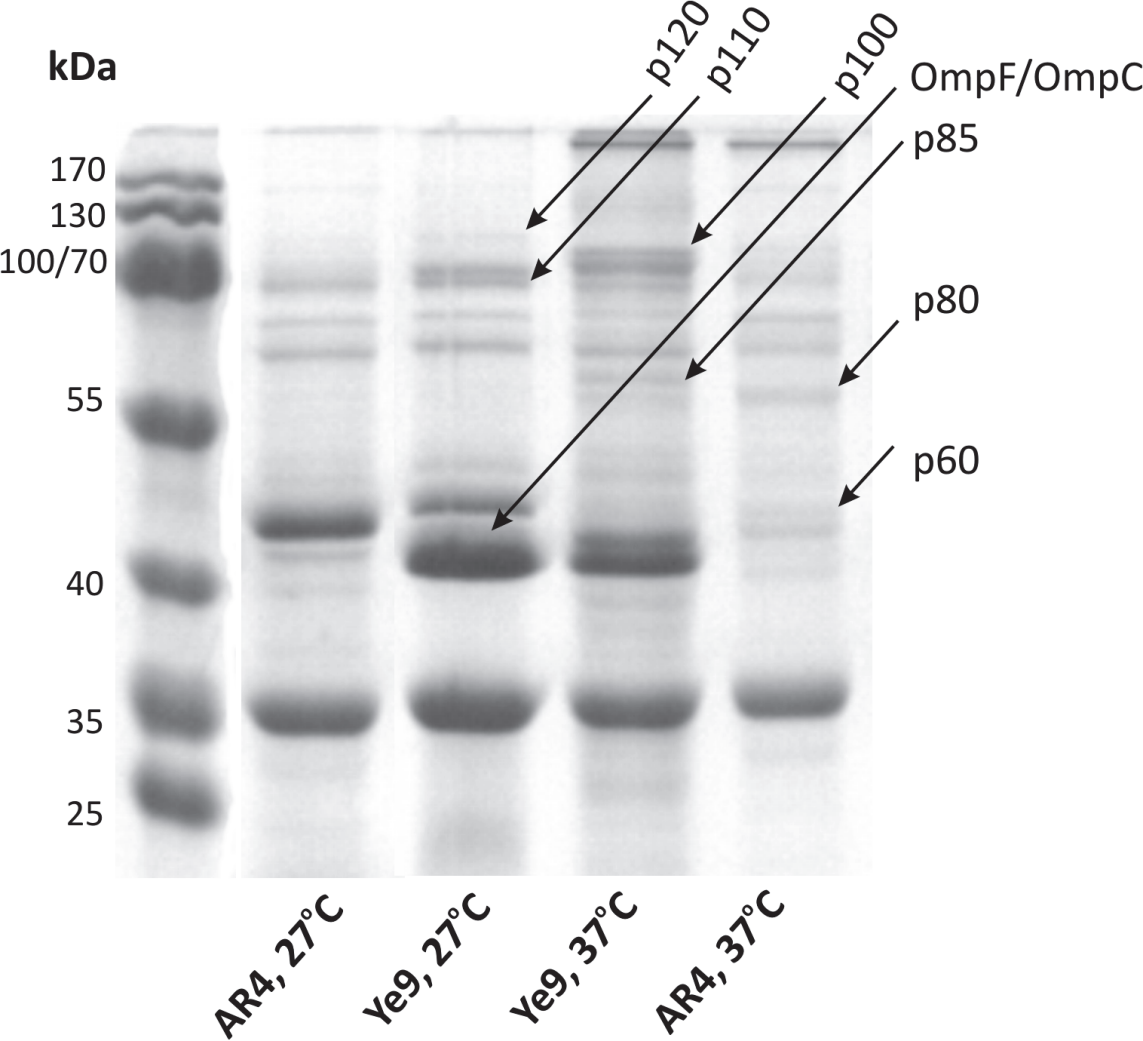
Influence of OmpR activity on the *Y*. *enterocolitica* membrane protein profile. Proteins were isolated from strains Ye9 (wild type) and AR4 (*ompR* mutant) grown overnight in LB medium at 27°C or 37°C. In each case, 50 μg of protein were separated by SDS-PAGE and visualized by Coomassie blue staining. Putative OmpR-regulated proteins subsequently identified by LC-MS/MS are indicated by arrows. The bands were named according to their migration in the 12% polyacrylamide gel relative to the molecular weight standards.

In parallel with the mutant screen described above, we analyzed membrane proteins of the wild-type *Y*. *enterocolitica* Ye9 and strain AR4 (the *ompR* mutant), grown under different temperature conditions, using SDS-PAGE. Changes in the membrane protein profiles due to the growth temperature and the presence and activity of the OmpR regulator were observed (Fig 2). A differential analysis of band intensities was used to select proteins for identification by LC-MS/MS. Following this procedure, we identified six putative OmpR-controlled proteins that matched *Y*. *enterocolitica* proteins present in the NCBI protein databases (Table 2). The synthesis of four proteins was higher in the wild-type strain than in mutant AR4: p120 and p110 (cells grown at 27°C), and p100 and p85 (cells grown at 37°C). Two other proteins, p80 and p60, were present at lower levels in the wild-type strain compared to AR4. The proteins that appeared to be positively controlled by OmpR were AcrB (p120; an essential component of the AcrAB-TolC multidrug efflux pump), ferrichrome-iron receptor (p110), dehydratase (p100; a bifunctional aconitate hydratase 2/2-methylisocitrate dehydratase) and a putative ABC transporter ATP-binding protein (p85). The proteins presumed to be negatively regulated by OmpR were a 60-kDa heat shock protein (p80) and elongation factor Tu (p60).

### Genes *acrR* and *dsrE* are organized in an operon

**Fig 3:**
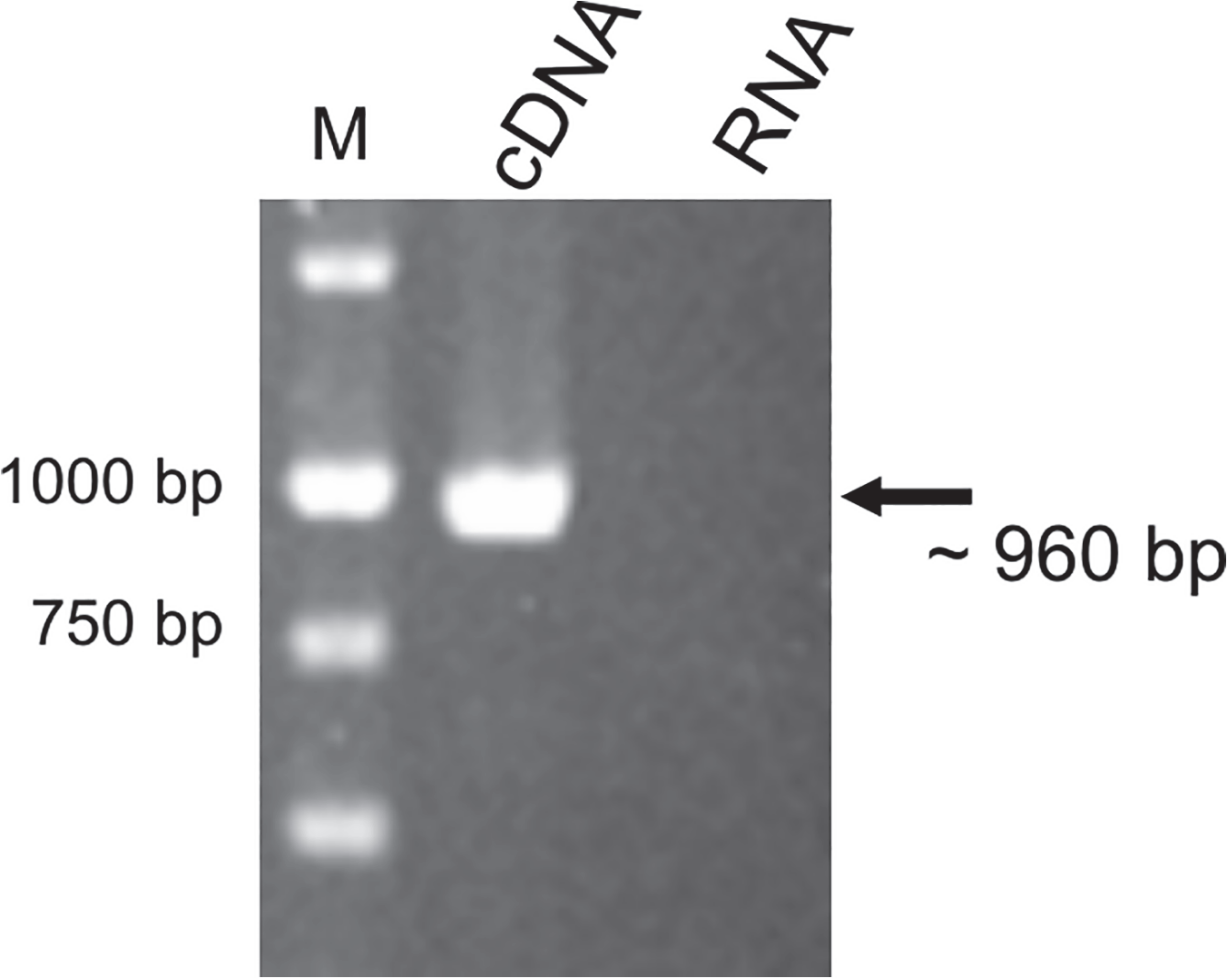
RT-PCR analysis of the *acrRdsrE* operon. The analysis was performed using total RNA isolated from strain Ye9 grown in LB medium at 27°C. The size of the amplified fragment was estimated by comparison with the size marker DNAs (lane M). A negative control reaction was performed using DNase-treated RNA as the template.

Transposition of Tn5-B22 in the strain AR83 (negative effect of OmpR) occurred in gene YE105_C1163 (encoding a DsrE homolog) located upstream of gene YE105_C1162, encoding transcriptional repressor AcrR. To determine whether these two genes are organized in an operon, RT-PCR analysis was performed (Fig 3). Our data showed that the putative *acrR* and *dsrE* genes of *Y*. *enterocolitica* Ye9 are co-transcribed as a bicistronic mRNA. Thus, the gene encoding transcriptional repressor AcrR seems to be negatively regulated by OmpR.

### Influence of OmpR activity on *acrR* and *acrAB* promoter function

**Fig 4:**
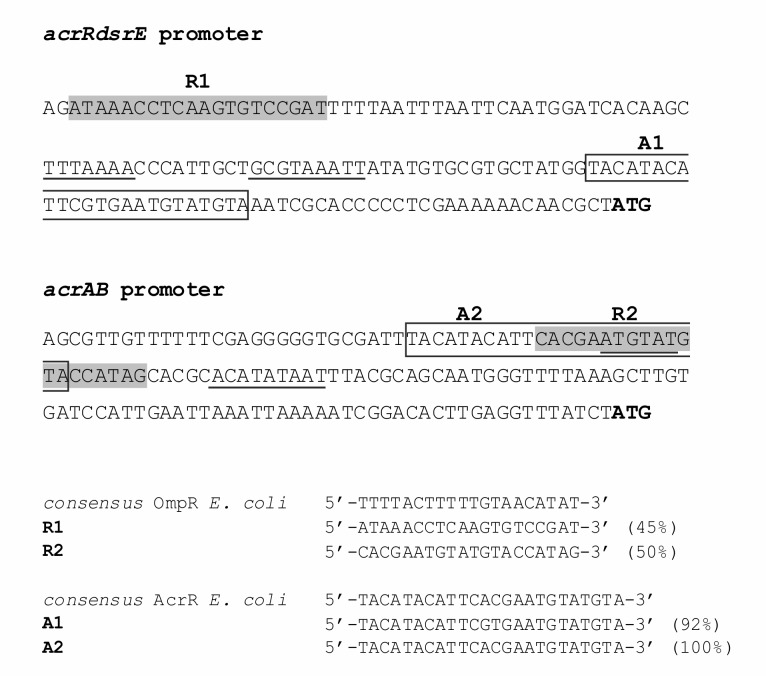
Intergenic sequences upstream of the *Y*. *enterocolitica acrR* and *acrAB* operons. The initiation codons (ATG) are in bold, and putative -35 and -10 promoter elements are underlined. The putative OmpR binding sites identified by *in silico* analysis (R1, R2) are shaded gray. Potential binding sites for AcrR (A1, A2) are boxed. Potential binding site elements aligned with the *E*. *coli consensus* sequences, with the percentage identities presented in parentheses.

**Fig 5:**
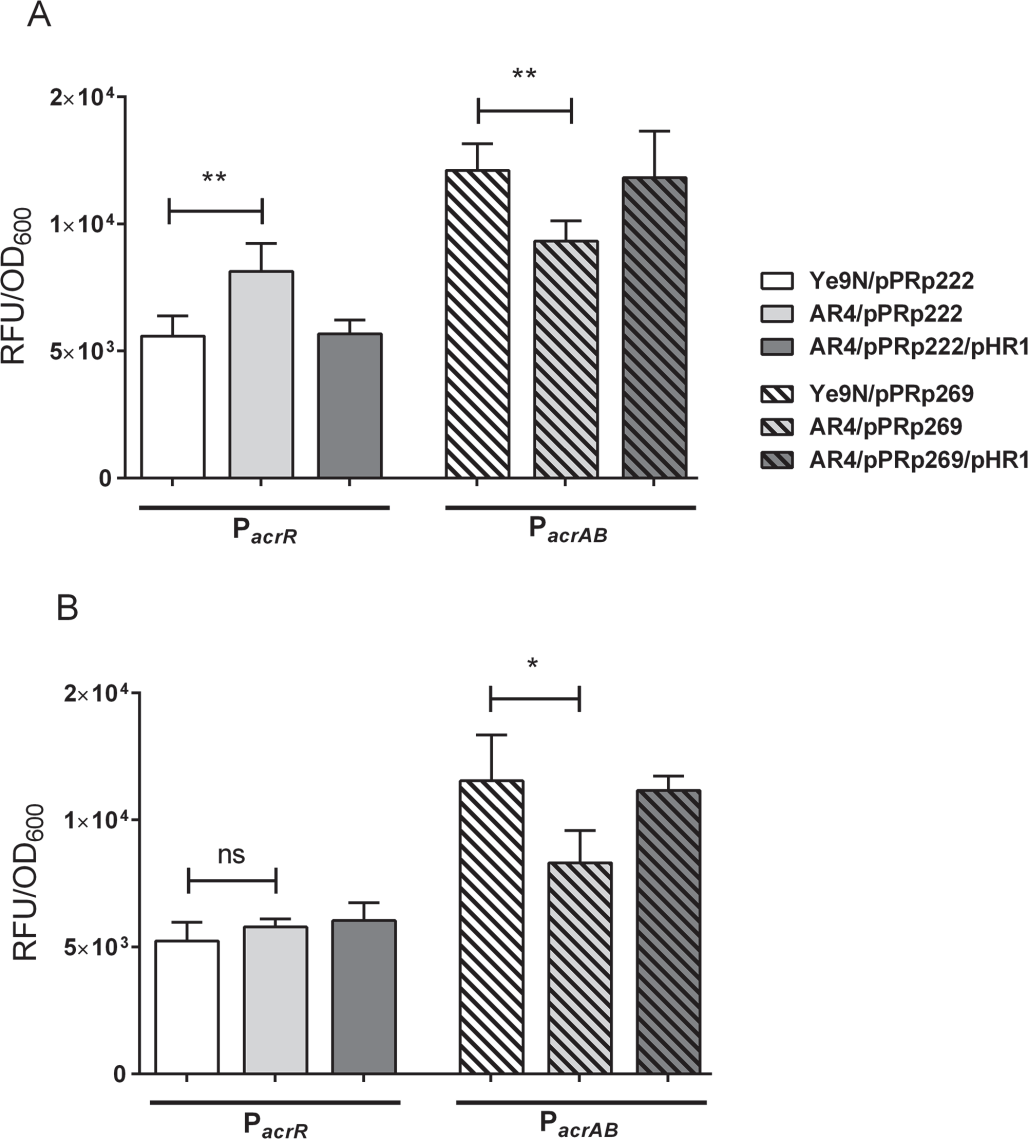
Effect of OmpR and growth phase on *acrR* and *acrAB* promoter activity. Strains Ye9 and AR4 carrying the promoter-*gfp* fusion constructs were cultivated in LB medium at 27°C to exponential phase (A) and stationary phase (B). Data represent mean fluorescence activity values normalized to the OD_600_ of the culture (± standard deviation) from three independent experiments performed in triplicate. The significance of differences between the values was calculated using Student’s unpaired *t*-test (ns [non significant]—*p*>0.05, *—*p*<0.05, **—*p*<0.01).

The genetic screen and the differential analysis of membrane proteins resulted in the identification of several putative members of the OmpR regulon. In one case, both of these methods suggested that OmpR might be involved in controlling expression of the AcrAB-TolC multidrug efflux pump in *Y*. *enterocolitica* Ye9. To confirm this hypothesis, we decided to construct fusions of the *acrR* and *acrAB* promoters with the *gfp* reporter gene. Bioinformatic analysis of the *acrR* and *acrAB* promoter regions revealed the presence of single putative OmpR binding sites, located at the respective positions -142 to -123 nt and -106 to -87 nt relative to the ATG start codons (Fig 4). These sequences exhibited 45% and 50% identity to the *E*. *coli* consensus OmpR-binding site, respectively, and both possessed the conserved motif GxxxC and the AC base pairs thought to be important for interaction with the OmpR regulator [36,37,38,39]. Taking into account the results of this *in silico* sequence analysis, reporter fusions of the *acrR* promoter (pPRp222, *acrR*::*gfp*) and *acrAB* promoter (pPRp269, *acrAB*::*gfp*) with *gfp* were constructed. The plasmid constructs carrying the appropriate fusions, i.e. pPRp222 and pPRp269, were then transformed into wild-type strain Ye9 and *ompR* mutant AR4, and GFP fluorescence was measured in bacteria grown in LB medium to exponential and stationary phase. Complementation analysis was performed with strains AR4/pPRp222 and AR4/pPRp269 carrying additional plasmid pHR4 encoding the active OmpR protein [29]. Data presented in Fig 5A shows that in the exponential phase of growth, the *ompR* mutant AR4 displayed a 30% increase in fluorescence produced by the *acrR*::*gfp* fusion (pPRp222) and a 20% decrease in fluorescence from the *acrAB*::*gfp* fusion (pPRp269), compared to the wild-type strain. In the stationary phase of growth, the lack of the OmpR protein resulted in a 30% decrease in the level of fluorescence activity of strain AR4/pPRp269, while no difference was observed for strain AR4/pPRp222, compared to the wild type (Fig 5B). These results demonstrated the involvement of OmpR in the negative regulation of *acrR* and the positive regulation of *acrAB* expression, depending on the growth phase.

### Effect of temperature and OmpR activity on *acrR* and *acrAB* promoter function

**Fig 6:**
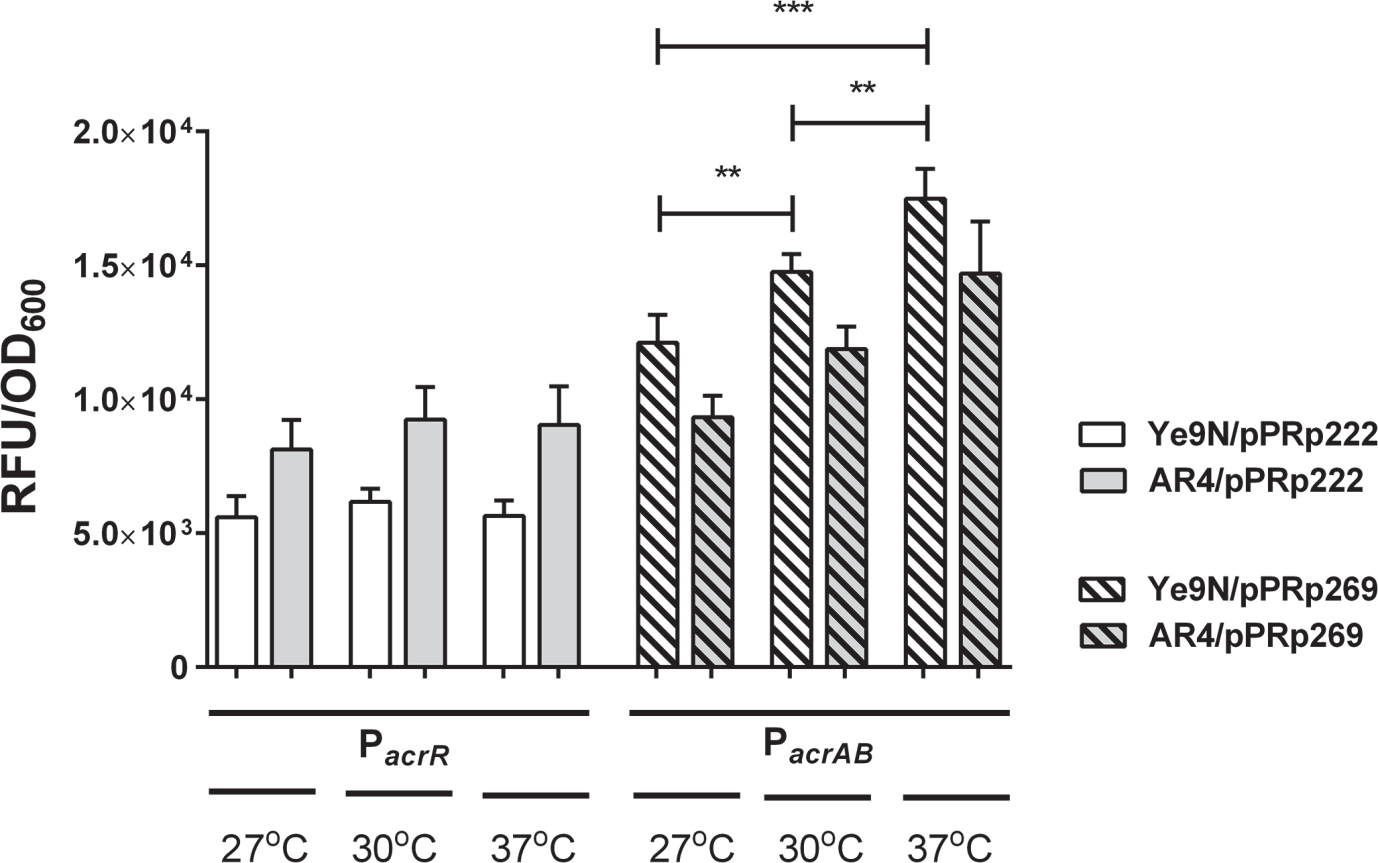
The influence of growth temperature on *acrR* and *acrAB* promoter activity. Strains Ye9 and AR4 carrying the promoter-*gfp* fusion constructs were cultivated in LB medium at 27°C, 30°C or 37°C to exponential phase. Data represent mean fluorescence activity values normalized to the OD_600_ of the culture (± standard deviation) from three independent experiments performed in triplicate. Significance of differences between the values was calculated using Student’s unpaired *t*-test (**—*p*<0.01, ***—*p*<0.001).

To study the influence of OmpR and temperature on the expression of *acrR* and *acrAB*, strains Ye9 and AR4 carrying pPRp222 or pPR269, were grown in LB medium at 27, 30 or 37°C to exponential phase, and the level of GFP fluorescence was measured (Fig 6). Compared with the level at 27°C, the GFP fluorescence produced by the *acrAB*::*gfp* fusion in wild-type strain Ye9 was increased by about 20% at 30°C, and by about 30% at 37°C (Fig 6). Thermoregulation of the *acrAB*::*gfp* fusion was also observed in *ompR* mutant strain AR4, although a relatively lower level of *acrAB* expression was noted. No differences in the level of GFP fluorescence were observed in either strain carrying the *acrR*::*gfp* fusion. These results indicated that temperature does not affect *acrR* expression, but is an important factor in regulating *acrAB* promoter activity, regardless of the presence or absence of the OmpR protein. Thus, OmpR is not involved in the observed thermoregulation.

### Effect of stress conditions on *acrR* and *acrAB* promoter function

Since the transcription of *acrAB* in *E*. *coli* and *Salmonella* is up-regulated by general stress factors, including the presence of bile salts, deoxycholate, indole, ethanol, paraquat, NaCl, and fluoroquinolones [8,13,40], we investigated the effect of these chemical stressors, and also low pH and the combination of high osmolarity/low pH, on *acrR* and *acrAB* expression using the *gfp* fusion constructs in the wild-type and *ompR* mutant strains (Table 3). Bile salts were found to be the most potent inducer of *acrR* and *acrAB* expression, causing an increase of approximately 3-fold in GFP fluorescence within 60 min of exposure. Interestingly, upregulation of *acrAB* expression by bile salts was found not to be OmpR-dependent. Deoxycholate, indole and paraquat only slightly modulated *acrAB*::*gfp* fluorescence activity in comparison to the control. None of the other tested chemicals affected the expression level of the analyzed *gfp* fusions. Moreover, no influence of osmolarity or pH was observed.

**Table 3:** Modulation of *acrR* and *acrAB* expression in strains Ye9N and AR4 by different stressors.

Strain	without	ciprofloxacin	sodium	ethanol	indole	paraquat	NaCl	pH 5.0	pH 5.0/ NaCl	bile salts
	stressors	(0.01 mM)	deoxycholate	(4%)	(3 mM)	(1 mM)	(300 mM)		(300 mM)	(5%)
			(0.5 mM)							
Ye9N/pPRp222^a^	5592 ± 395	4959 ± 318 (ns)	5913 ± 363 (ns)	5444 ± 748 (ns)	6086 ± 484 (ns)	4535 ± 351 (ns)	5270 ± 246 (ns)	5902 ± 654 (ns)	6232 ± 534 (ns)	15927 ± 1680 (***)
AR4/pPRp222^b^	8130 ± 552	5674 ± 161 (*)	6564 ± 459 (ns)	5889 ± 266 (*)	6609 ± 326 (ns)	5700 ± 353 (*)	6513 ± 405 (ns)	6979 ± 367 (ns)	6611 ± 345 (ns)	33721 ± 1389 (***)
AR4/pPRp222/pHR4	5675 ± 383	5274 ± 221	5381 ± 674	5546 ± 138	5925 ± 172	4723 ± 443	5645 ± 346	6108 ± 344	6571 ± 452	22078 ± 1650
Ye9N/pPRp269^c^	12107 ±523	10882 ± 921 (ns)	15461 ± 786 (**)	11931 ± 475 (ns)	14562 ± 456 (*)	14225 ± 756 (*)	10538 ± 680 (ns)	13493 ± 832 (ns)	12765 ± 820 (ns)	39100 ± 1073 (***)
AR4/pPRp269^d^	9329 ± 400	8359 ± 860 (ns)	10007 ± 641 (ns)	9227 ± 389 (ns)	10445 ± 375 (ns)	10060 ± 540 (ns)	8440 ± 385 (ns)	9947 ± 412 (ns)	9174 ± 512 (ns)	35379 ± 1016 (***)
AR4/pPRp269/pHR4	11829 ± 1051	10391 ± 631	13016 ± 502	11112 ± 614	14293 ± 363	13894 ± 469	11513 ± 832	14006 ± 387	13984 ± 784	39398 ± 1370

The GFP fluorescence (RFU/OD_600_) produced by the *acrR*::*gfp* (pPRp222) and *acrAB*::*gfp* (pPRp269) fusions in Ye9N and AR4 strains are presented. Data shown are mean values ± standard deviation from three independent experiments performed in triplicate. Significance was calculated with Student’s unpaired *t*-test [ns (non significant)—*p*>0.05

*—*p*<0.05

**—*p*<0.01

***—*p*<0.001].

^a, c^ Results for Ye9N strains, grown in LB medium without any stressors, were compared with those for equivalent Ye9N strains, grown in the presence of different stressors.

^b, d^ Results for AR4 strains were compared with those for equivalent Ye9N strains grown in the same conditions.

### Interaction of OmpR with the *acrR* and *acrAB* promoter regions

**Fig 7:**
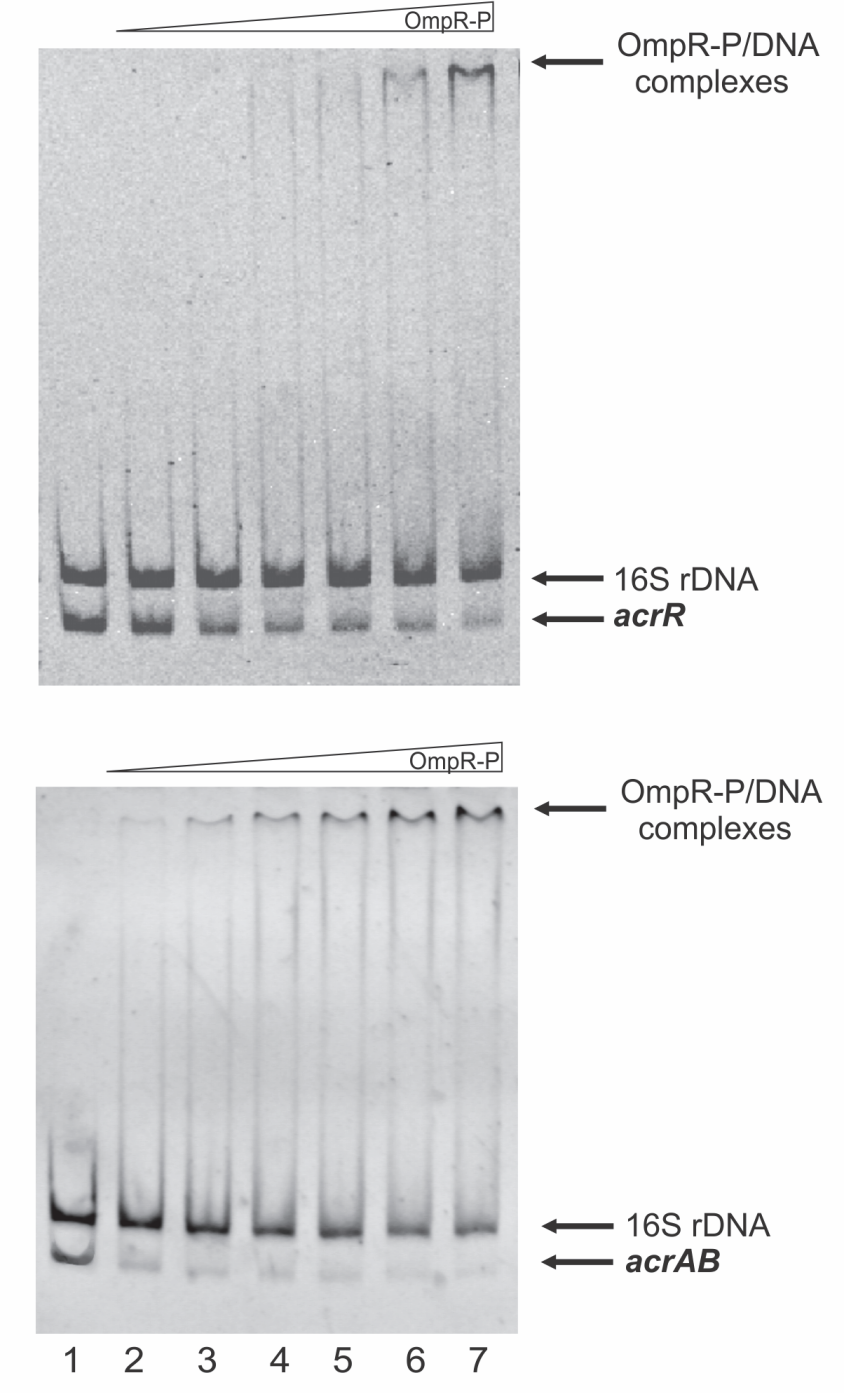
Interaction of purified OmpR with the *acrR* and *acrAB* promoter regions examined by electrophoretic mobility shift assays. EMSAs using *Y*. *enterocolitica acrR* (upper panel) or *acrAB* (lower panel) promoter fragments with *in vitro* phosphorylated OmpR protein (lane 1—no protein; lane 2–1.2 μM; lane 3–2.5 μM; lane 4–3.7 μM; lane 5–5 μM; lane 6–6.2 μM; lane 7–7.5 μM). A 16S rDNA fragment was included as a control in all reactions to confirm specific binding.

To test whether OmpR interacts with the *acrR* and/or *acrAB* promoter regions to directly control expression, we performed electrophoretic mobility shift assays (EMSAs). A recombinant OmpR-His_6_ protein was expressed in *E*. *coli* and purified to homogeneity. Since the affinity of OmpR for targets is dependent on its phosphorylation status, gel shift assays were performed in the presence of acetyl-phosphate to promote OmpR auto-phosphorylation. As mentioned above, one putative OmpR binding site (-142 to -123 nt from the ATG) and also one putative AcrR motif (-52 to -29 nt from the ATG) were recognized in the *acrR* promoter region. Moreover, a putative binding site for OmpR (-106 to -87 nt from the ATG), overlapped the putative AcrR binding motif (-116 to -93 from the ATG) and -35 promoter motif in the *acrAB* promoter region (Fig 4). Thus, fragments containing these putative binding sites within the *acrR* promoter (p222) and *acrAB* promoter (p269) were used as the target DNAs in EMSAs. Different amounts of the purified OmpR were incubated with these promoter fragments and these binding reactions were analyzed by electrophoresis in 6% non-denaturing polyacrylamide gels (Fig 7). Shifted complexes were clearly produced when the *acrAB* p269 fragment interacted with the phosphorylated OmpR protein present at the lowest concentration, i.e. 1.2 μM (Fig 7, lower panel). Furthermore, a slower migrating band also appeared when a higher amount of phosphorylated OmpR (6.2 μM) was incubated with the *acrR* p222 fragment (Fig 7, upper panel), but this interaction seemed weaker. The observed interactions were specific because no OmpR-P binding of a 16S rDNA control fragment was observed. Interestingly, non-phosphorylated OmpR was found to interact with the two promoter fragments with the same affinity as the phosphorylated form (data not shown). Taken together, these results demonstrated that OmpR, regardless of its phosphorylation state, can specifically bind to the *acrR* and *acrAB* promoter regions with different affinity, to inhibit and activate transcription, respectively.

## Discussion

The success of *Y*. *enterocolitica* as a pathogen depends on its rapid and efficient response to changing environmental conditions. TCSs play a significant role in sensing external factors and modulating the transcription of specific genes accordingly. It seems that OmpR could operate as a global regulatory protein participating in the control of many genes, including virulence determinants. To identify new members of the OmpR regulon, we performed a genetic screen and a differential analysis of membrane proteins. Initially, we employed transposon Tn5-B22 carrying a promoterless *lacZ* reporter gene to produce 1000 insertion mutants and studied their phenotypes in the presence and absence of OmpR. We recognize that the pool of mutants examined in this study only represents a relatively small sample of possible transposon insertions. The *Y*. *enterocolitica* subsp. *palearctica* 105.5R(r) genome is 4.50 Mb in size and carries 4441 genes. Together with plasmid pYV, characteristic of pathogenic *Yersinia* spp., which contains 110 genes, this species possesses about 4600 genes in total, so 5000 transposon mutants would correspond to 1x ORF coverage of the genome. Thus, our transposon library only covered a fraction of these ORFs, which implies that additional genes subject to regulation by OmpR remain unrecognized. On the basis of differences in *β-*galactosidase activity, 7 mutants were identified with insertions in genes/operons positively and negatively regulated by OmpR. A particularly noteworthy mutant was strain AR83, which exhibited an 8-fold decrease in *lacZ* expression in the presence of OmpR *in trans*. For the other mutants, these differences in *β-*galactosidase activity were considerably smaller. Genetic analysis of mutant AR83 revealed that transposition had occurred in a gene encoding a hypothetical protein with homology to DsrE, a protein essential to oxidative sulfur metabolism [41]. Interestingly, our *in silico* analysis revealed that immediately upstream of *dsrE* in the *Y*. *enterocolitica* genome is a gene encoding transcriptional repressor AcrR, involved in the regulation of *acrAB* expression in *Enterobacteriaceae* [42]. The AcrA and AcrB proteins are components of the multidrug efflux pump AcrAB-TolC [5]. In *E*. *coli* the *acrAB* operon is located upstream of the *acrR* gene and transcribed in the opposite orientation. In the course of the present study we demonstrated that the *acrR* and *dsrE* genes are cotranscribed from one promoter, and this gene pair constitutes an operon in *Y*. *enterocolitica* Ye9. This organization of the *acrR* gene seems to be characteristic only for the genus *Yersinia*, as judged by *in silico* analysis (data not shown). A differential analysis of *Y*. *enterocolitica* membrane proteins performed in parallel with the genetic screen revealed that apart from the OmpC/F porins, OmpR is required for the expression of at least six other proteins. We identified these putative OmpR-controlled proteins by SDS-PAGE followed by LC-MS/MS analysis. Among these, the protein AcrB was subject to positive control involving OmpR. This finding was consistent with the result of the genetic screen which identified *dsrE* and the co-transcribed *acrR* gene, encoding repressor AcrR, as being subject to negative OmpR control. In the light of these data, it seems that OmpR might be involved in regulating the AcrAB-TolC multidrug efflux pump directly, or indirectly via repressor AcrR. Thus, the role of OmpR in the mechanism controlling *acrR* and *acrAB* expression became the subject of subsequent experiments. We first examined the effect of the *ompR* mutation on the transcription of *acrR* and *acrAB* using promoter-*gfp* reporter fusions. By monitoring the activity of the *acrR*::*gfp* and *acrAB*::*gfp* fusions in strains Ye9 and AR4, we showed that OmpR plays an important role in the inhibition of *acrR* expression and the induction of *acrAB* expression in the exponential phase of bacterial growth. Interestingly, we observed a clear effect of temperature on *acrAB* expression (but not on that of *acrR*), which has not been observed in other enterobacteria. It is known that the EnvZ/OmpR TCS is stimulated by environmental conditions such as osmolarity or low pH, and it regulates many genes of diverse function [43]. Our results demonstrated that low pH, high osmolarity or a combination of both stress conditions did not influence *acrR* or *acrAB* expression. Of the chemical stressors tested for their influence on *acrR* and *acrAB* expression, only bile salts were found to cause the induction of both, which is in agreement with previous studies on *S*. Typhimurium and *E*. *coli* [8,13,40]. However, this effect was independent of the OmpR regulator.

Many bacterial operons are under the control of both global and specific regulators. It is known that apart from AcrR, the expression of *acrAB* is influenced by four other regulators: MarA, SoxS, Rob and RamA [8,9,10,12,13]. The induction of *acrAB* in *Salmonella* mediated by RamA occurs in response to indole and bile salts. The Rob protein is activated by bile salts and causes increased expression of the *mar* operon [44]. Furthermore, the superoxide generator paraquat activates *acrAB* via SoxS [45]. Other stress signals, including ethanol and osmolarity, also upregulate *acrAB*, but independently of the activity of regulators MarA, Rob or SoxS [8]. In *Y*. *pestis*, MarA mediates overexpression of *acrAB*, contributing to multidrug resistance [46]. MarA is a member of the XylS/AraC family of transcriptional activators like Rob and SoxS [47]. Our *in silico* analysis of the *Y*. *enterocolitica* genome indicated that this bacteria possesses orthologs of MarA, Rob and SoxS, but not RamA. These findings suggest the participation of MarA or other factors in *acrAB* and *acrR* expression in *Y*. *enterocolitica* in response to bile salts, and highlight the complexity of the mechanism that modulates the transcription of these operons. We have yet to identify the factor(s) that act to induce the OmpR-mediated expression of *acrR* or *acrAB*. It appears that the AcrR regulon belongs to a “stress-regulated” network, where genes are under the control of a number of apparently independent environmental stimuli. Bacteria experience the continual fluctuation of many environmental parameters, which requires fine tuning of the expression of many genes including *acrR* and *acrAB*. This would explain the difficulty in identifying a single factor mediating changes in the expression level of genes such as these. It appears that as *Y*. *enterocolitica* moves from the external environment into the intestinal tract, the changing conditions, such as the temperature shift to 37°C and raised levels of toxic compounds, especially bile salts, contribute to the induction of AcrAB.

To gain further insight into the regulatory mechanism of *acrR* and *acrAB* expression involving OmpR, we performed electrophoretic mobility shift assays. Prior *in silico* analysis identified single putative OmpR-binding sites in the promoters of these operons, similar to the conserved OmpR binding site reported in *E*. *coli* [36,37,38,39]. The deduced amino acid sequence of OmpR from *Y*. *enterocolitica* Ye9 is 99% identical to *E*. *coli* OmpR (data not shown) and exhibits 98% similarity to the OmpR of *S*. Typhimurium [48]. This suggests that binding sites for this regulator are also highly similar in these bacteria. EMSAs performed using OmpR or phosphorylated OmpR (OmpR-P) revealed specific binding of both forms to *acrR* and *acrAB* promoter fragments, with higher affinity for the latter. Previously it has been demonstrated that non-phosphorylated OmpR can bind the promoter of the *fimB* gene of uropathogenic *E*. *coli* (UPEC). It was also shown that the OmpR protein level could be responsible for the regulation of *fimB* in this bacterium [49]. Thus, it seems that apart from the phosphorylation state, the level of OmpR protein in *Y*. *enterocolitica* cells might influence *acrR* and *acrAB* expression.

The likely OmpR binding site in the *acrAB* promoter region overlaps the AcrR binding site, which suggests an antirepressor-type role for OmpR in the transcriptional regulation of this operon. Thus, the results of this study indicate that *Y*. *enterocolitica acrAB* might be subject to dual regulation by OmpR. We suggest that the transcription of *acrR* and *acrAB* are both directly OmpR-dependent, and OmpR also controls *acrAB* expression indirectly through AcrR via a locally limited feedback loop.

The molecular mechanism of OmpR-dependent regulation is not fully understood [39]. There is strong evidence that OmpR in *Salmonella* not only acts as a classical site-specific transcription factor, that activates both the SPI-1 and SPI-2 promoters (*Salmonella* pathogenicity island-1 and -2) through RNAP interaction, but it can also be considered a chromosome-structuring protein. The relaxation of DNA supercoiling can both induce and repress gene expression [50,51]. It has been shown that stress-induced changes in DNA topology recruit OmpR to the promoters of *Salmonella* genes *ssrA*, *hilC* and *hilD*, encoding transcriptional regulators of SPI-1 [52]. It is possible that this form of regulation, based on DNA topology, together with OmpR level or activity, could coordinate the expression of *acrAB* and/or *acrR* in *Y*. *enterocolitica* in response to environmental stimuli.

## Materials and Methods

### Bacterial strains, plasmids, media and growth conditions

The strains and plasmids used in this work are listed in Table 4. *E*. *coli* and *Y*. *enterocolitica* strains were routinely grown under aerobic conditions at 27 or 37°C in LB (Luria-Bertani) broth or on LB agar plates. When necessary, media were supplemented with the following compounds: nalidixic acid (Nal) – 30 μg ml^−^1; kanamycin (Km) – 50 μg ml^−1^; gentamicin (Gm) – 45 μg ml^−1^; chloramphenicol (Cm) – 25 μg ml^−1^; tetracycline (Tet) – 12.5 μg ml^−1^; X-Gal (5-bromo-4-chloro-3-indolyl galactoside) – 80 μg ml^−1^.

**Table 4:** Strains and plasmids used in this study.

Strain or plasmid	Description	Reference or source
***Y*. *enterocolitica* O9**		
Ye9	pYV^+^, wild type	Laboratory
		collection
AR4	pYV^+^, Δ*ompR*::Km (Nal^R^, Km^R^)	[29]
AR22,AR24, AR33	pTn*5*-B22 derivatives of AR4 (Nal^R^, Km^R^,	This study
AR53, AR83	Gm^R^)	
AR202, AR704		
***E*. *coli***		
Top10 F’	F’{*lacI* ^*q*^ Tn10 (Tet^R^)} *mcrA* Δ(*mrr-hsd RMS-*	Invitrogen
	*mcrBC*) *ϕ80lacZ* Δ*M15* Δ *lacX74 deoR recA1*	
	*araD139* Δ*99ara-leu*)7697	
**Plasmids**		
pDrive	cloning vector (Ap^R^, Km^R^)	Qiagen
pSUP101Tn5-B22	mobilizable suicide vector pSUP101 derivative	[59]
	with Tn5 element containing promoterless	
	*lacZ* (Tc^R^, Gm^R^)	
pBR3	pBBR1 MCS-3 with XhoI/PstI fragment	[30]
	containing entire coding sequence of *ompR*	
	(Tc^R^)	
pHR4	pHSG575 with 740-bp fragment of *ompR* (ORF	[29]
	with rbs) (Cm^R^)	
pPROBE TT’	broad-host-range cloning vector pBBR1MCS-3	[57]
	with promoterless *gfp* (Tc^R^)	
pPRp222	derivative of pPROBE TT’ with EcoRI/KpnI	This study
	fragment of *acrRdsrE* promoter region (Tc^R^)	
pPRp269	derivative of pPROBE TT’ with EcoRI/KpnI	This study
	fragment of *acrAB* promoter region (Tc^R^)	

To determine the influence of different stress factors on gene expression, various compounds, including an antibiotic (ciprofoxacin), biocide (paraquat), and other chemicals (indole, deoxycholate, bile salts, ethanol), were used at sub-inhibitory concentrations [53]. Sodium chloride (300 mM NaCl) was used to induce osmotic shock. To monitor the influence of low pH on gene expression, we buffered LB medium to pH 5.0 with 0.1 M homoPIPES [homopiperazine-*N*,*N’*-bis(2-ethanesulfonic acid)].

### Random insertion of the *lacZ* reporter gene throughout the genome of *Y*. *enterocolitica ompR* mutant strain AR4 (Δ*ompR*::Km) followed by *trans*-complementation with a wild-type copy of *ompR*

Transposon mutagenesis of the *Y*. *enterocolitica ompR* mutant with Tn5-B22 (carrying the promoterless *lacZ* reporter gene) was performed using suicide plasmid pSUP101Tn5-B22 according to the procedure described by Robleto *et al*. [54]. Clones carrying the randomly inserted transposon were selected on LB agar plates containing appropriate antibiotics. Next, the individual transposon mutants were patched in 12 x 8 grids onto LB agar plates and then transferred, using 96-prong replicators into microtiter plates containing LB broth. Plasmid pBR3 carrying a wild-type copy of *ompR* was introduced *in trans* into each of the mutants by the following mass-mating technique. *E*. *coli* S17-1 λ*pir/*pBR3 was grown overnight in LB broth supplemented with Tet. The cells were pelleted by centrifugation, washed twice with LB broth to remove antibiotics, and resuspended in the original volume of LB. Aliquots of this cell suspension (200 μl) were spread onto LB agar plates to form a lawn of the donor strain. The *Y*. *enterocolitica* mutants were replicated onto this lawn of *E*. *coli* S17-1 λ*pir*/pBR3 and grown overnight at 27°C. These mating spots were then replicated onto LB agar plates containing appropriate antibiotics to select for transconjugants carrying plasmid pBR3. Collections of strains with and without pBR3 were replicated into 96-well microtiter plates containing LB broth with appropriate antibiotics and incubated overnight at 27°C. Individual transposon mutants were then screened to detect differences in reporter gene expression in the presence and absence of active *ompR* by growth on LB agar plates supplemented with X-Gal and antibiotics. Mutants that differed in the intensity of the blue color were identified, the corresponding patch was streaked onto a fresh plate, and a single colony was retested for *β*-galactosidase activity as confirmation.

### *β*-Galactosidase assays

*β*-galactosidase activities were assayed by the method of Miller [55] with ONPG (*o*-nitrophenyl-*β-*D-galactopyranoside) as the substrate. Assays were routinely performed at least three times in triplicate and the data expressed as the mean ± standard deviation.

### Arbitrary-primer PCR (AP-PCR) for amplification of DNA regions flanking transposon insertions

AP-PCR was carried out in two rounds, each with a different primer pair (S1 Table). In the first round, primer TN5EXT (sequence unique to the right end of the transposon) and an arbitrary primer ARB1 were used [54]. PCR was performed as follows: (i) 5 min at 95°C; (ii) six cycles of 30 s at 95°C, 30 s at 30°C, and 1 min 30 s at 72°C; (iii) 30 cycles of 30 s at 95°C, 30 s at 45°C, and 2 min at 72°C; and (iv) a final extension step of 5 min at 72°C. The second round of PCR was performed using 2 μl of the first round reaction as template with primers TN5INT (specific to the right arm of the transposon) and ARB3 (specific to the 5’ end of primer ARB1) [54]. PCR was performed as follows: (i) 1 min at 95°C; (ii) 30 cycles of 30 s at 95°C, 30 s at 52°C, and 2 min at 72°C; and (iii) a final extension step of 5 min at 72°C. The PCR products were separated by agarose gel electrophoresis and the most-intense bands were purified (Invitrogen PureLink Gel extraction kit) and sequenced using primers TN5INT and ARB3 with an ABI PRISM 377 DNA Sequencer (DNA Sequencing and Oligonucleotide Synthesis Laboratory, IBB PAN, Warsaw). The sequences were compared with sequence databases using BLAST programs from The National Center for Biotechnology Information (http://www.ncbi.nlm.nih.gov/).

### DNA techniques

DNA manipulations, such as restriction digestion, ligation, transformation and conjugation were performed using standard protocols [56]. Plasmid and chromosomal DNA were purified using the BlueMATRIX Plasmid Miniprep DNA kit and Gene MATRIX Bacterial Genomic +/- DNA Purification Kit (EURx), respectively. DNA fragments were amplified by PCR using Taq DNA polymerase (Invitrogen) and oligonucleotide primers (S1 Table). PCR products were purified from the reactions using a BlueMATRIX PCR/DNA Clean-up DNA Purification Kit or following agarose gel electrophoresis with the BlueMATRIX Agarose-Out DNA Purification Kit (EURx).

### Isolation of membrane proteins and SDS-PAGE analysis

Outer membrane protein-enriched fractions were obtained by treatment of *Y*. *enterocolitica* cell envelope samples with Triton X-100. These fractions, referred to henceforth as membrane proteins, were isolated from strains grown to late exponential phase at 27°C and 37°C as described previously [31]. The samples were resuspended in electrophoresis sample buffer, solubilized by boiling for 10 min and the proteins separated by electrophoresis on 12% SDS-polyacrylamide gels. Proteins were visualized by Coomassie Brilliant Blue R-250 staining.

Membrane proteins were isolated from *Y*. *enterocolitica* strains differing in their OmpR content, in three separate experiments, and SDS-PAGE of each protein preparation was performed twice. Only proteins that were clearly and reproducibly increased or decreased in relative abundance compared to the wild type strain, based on their band intensity, were further analyzed.

### Protein identification by liquid chromatography-coupled tandem mass spectrometry (LC-MS/MS)

Gel slices containing the appropriate bands, visualized by Coomassie Blue staining, were excised with a clean scalpel. Prior to the LC-MS/MS analysis, the gel slices were subjected to in-gel trypsin digestion [33]. LC-MS/MS analysis of proteins was performed at the Mass Spectrometry Laboratory, IBB PAN, Warsaw.

### Construction of *acrR*::*gfp* and *acrAB*::*gfp* transcriptional fusions

To obtain *acrR*::*gfp* and *acrAB*::*gfp* transcriptional fusions, appropriate DNA fragments containing the promoters of the *acrR* and *acrAB* operons were amplified by PCR using the oligonucleotide pairs FacR1/RacR222 and FacAB1/RacAB269, respectively. The promoter fragments of *acrR* (p222) and *acrAB* (p269) were cloned into the cloning vector pDrive (Qiagen). The fragments were then excised by digestion with EcoRI/KpnI and subcloned into the same sites of reporter vector pPROBE TT’ to place them adjacent to a promoterless *gfp* gene [57]. The resulting constructs pPRp222 and pPR269 were transferred to *Y*. *enterocolitica* strains Ye9 and AR4 by electroporation. Transformants were selected on LB agar plates supplemented with Tet (strain Ye9), or Tet and Km (strain AR4). The presence of these constructs in the *Y*. *enterocolitica* strains was confirmed by plasmid isolation and PCR with the primers used previously to amplify the promoter fragments.

### Measurement of GFP fluorescence

To test the effect of different stress factors on gene expression, early log-phase bacterial cultures (OD_600_ ~0.2) grown in LB medium were exposed to ciprofloxacin, ethanol, indole, paraquat, bile salts, deoxycholate, 300 mM NaCl, pH 5.0 or a combination of low pH/high osmolatity at 27°C for 60 min. To monitor the influence of temperature, identical cultures were grown in LB medium at 27°C, 30°C or 37°C for 2 h. Then, 200 μl of the treated cell suspensions were transferred to wells of 96-well flat-bottomed plates (Costar) and absorbance at 600 nm and GFP fluorescence (excitation 485 nm; emission 530 nm) were measured using a TECAN infinite pro M200PRO microplate reader, according to the procedure described by Gueguen *et al*. [58]. The specific GFP fluorescence was expressed as the relative fluorescence intensity (RFU) divided by the OD_600_, after subtracting the values of a blank sample. A culture of strain Ye9 carrying vector pPROBE TT’ was used to determine background fluorescence. Individual cultures were assayed in triplicate and the reported values are the means from three independent cultures.

### RNA isolation and reverse transcription

Total RNA was isolated from 10^7^ bacterial cells from cultures grown overnight in LB medium at 27°C, using a BlueMATRIX Universal RNA Purification Kit (EURx). Following treatment with RNase-free DNase I (Sigma), RNA was reverse-transcribed with random hexamers and AMV reverse transcriptase (Sigma). The cDNA was used as the template in PCRs (RNA used as a negative control) with primers RTacR1 and RTdrE2 (S1 Table) specific for the *acrRdsrE* mRNA. The amplified fragment was resolved by electrophoresis on 2% agarose gels and visualized by staining with ethidium bromide.

### Electrophoretic mobility shift assays (EMSAs)

OmpR binding studies were performed using purified N-terminal His-tagged OmpR protein and DNA fragments containing the *acrR* and *acrAB* control regions. The *acrR* promoter fragment p222 used in the transcriptional fusion with *gfp* was employed in the EMSA. However, a new *acrAB* promoter fragment p225 was amplified for this purpose by PCR with primer pair FacAB1/RacAB225 (S1 Table). OmpR-His_6_ synthesized in *E*. *coli* M15 was purified using Ni^2+^-NTA agarose as described previously [30]. The EMSA method was adapted from a previously published protocol [31]. For phosphorylation of OmpR, 20 mM acetyl phosphate (Sigma) was used. To confirm binding specificity, a 304-bp fragment of *Y*. *enterocolitica* Ye9 16S rDNA generated by PCR using primer pair 16SR1/16SR304 (S1 Table) was included as a non-specific competitor in all binding reactions. The reaction mixtures were analyzed by electrophoresis on 6% non-denaturing polyacrylamide gels run in TBE buffer (Tris-borate-EDTA) and DNA bands were stained with SYBR Green EMSA nucleic acid gel stain (Invitrogen).

## Supporting Information

S1 Table Oligonucleotides used in this study. (PDF)
