# Supplementary material for: Expression of the AcrAB Components of the AcrAB-TolC Multidrug Efflux Pump of Yersinia enterocolitica Is Subject to Dual Regulation by OmpR
Source: PLoS One. 2015 Apr 20;10(4):e0124248. doi: 10.1371/journal.pone.0124248 (PMC4403819; doi:10.1371/journal.pone.0124248)
Supplement: S1 Table — (PDF) [file pone.0124248.s001.pdf]

S1 Table Oligonucleotides used in this study

purpose and target gene	name of primer	primer sequence
AP-PCR		
	TN5EXT	5’-GAACGTTACCATGTTAGGAGGTC-3’
	ARB1	5’-GGCCAGCCGTCGACTCANNNNNNNNNNGATA-3’
	TN5INT	5’-CGGGAAAGGTTCCGTTCAGGACGC-3’
	ARB3	5’-GGCCACGCGTCGACTAGTCA-3’
Promoter region and EMSA		
acrRdsrE	FacR1	5'-TGGAATTCATTGCAGCCAGAGGCATTAC-3'
	RacR222	5'-TGGGTACCTTGCTGTTTGGTTTTTCGTG-3'
acrAB	FacAB1	5'-TGGAATTCTAAAATTTGTTGCCGGGTCT-3'
	RacAB269	5'-TGGGTACCAGCTGCCTGAAAGTACCAGAA-3'
RT-PCR		
acrRdsrE	RTacR1	5'-ACCCGGCAACAAATTTTAGA-3'
	RTdrE2	5'-TTCAACCAAAGTGCCGATTT-3'
control EMSA		
16S rDNA	16SR1	5’-ATTCCGATTAACGCTTGCAC-3’
	16SR307	5’-GTGGGGTAATGGCTCACCTA-3’
